# KIF2C is a prognostic biomarker associated with immune cell infiltration in breast cancer

**DOI:** 10.1186/s12885-023-10788-4

**Published:** 2023-04-04

**Authors:** Shanshan Liu, Ziwei Ye, Vivian Weiwen Xue, Qi Sun, Huan Li, Desheng Lu

**Affiliations:** Guangdong Provincial Key Laboratory of Regional Immunity and Diseases, Department of Pharmacology, Carson International Cancer Center, Shenzhen University Medical School, Shenzhen, Guangdong 518055 China

**Keywords:** KIF2C, Breast carcinoma, Immune infiltration, Prognosis, Nomogram

## Abstract

**Background:**

The kinesin-13 family member 2C (KIF2C) is a versatile protein participating in many biological processes. KIF2C is frequently up-regulated in multiple types of cancer and is associated with cancer development. However, the role of KIF2C in immune cell infiltration of tumor microenvironment and immunotherapy in breast cancer remains unclear.

**Methods:**

The expression of KIF2C was analyzed using Tumor Immune Estimation Resource (TIMER) database and further verified by immunohistochemical staining in human breast cancer tissues. The correlation between KIF2C expression and clinical parameters, the impact of KIF2C on clinical prognosis and independent prognostic factors were analyzed by using TCGA database, the Kaplan-Meier plotter, and Univariate and multivariate Cox analyses, respectively. The nomograms were constructed according to independent prognostic factors and validated with C-index, calibration curves, ROC curves, and decision curve analysis. A gene set enrichment analysis (GSEA) was performed to explore the underlying molecular mechanisms of KIF2C. The degree of immune infiltration was assessed by the Estimation of Stromal and Immune cells in Malignant Tumor tissues using the Expression (ESTIMATE) algorithm and the single sample GSEA (ssGSEA). The Tumor mutational burden and Tumor Immune Dysfunction and Rejection (TIDE) were used to analyze immunotherapeutic efficiency. Finally, the KIF2C-related competing endogenous RNA (ceRNA) network was constructed to predict the putative regulatory mechanisms of KIF2C.

**Results:**

KIF2C was remarkably up-regulated in 18 different types of cancers, including breast cancer. Kaplan-Meier survival analysis showed that high KIF2C expression was associated with poor overall survival (OS). KIF2C expression was associated with clinical parameters such as age, TMN stage, T status, and molecular subtypes. We identified age, stage, estrogen receptor (ER) and KIF2C expression as OS-related independent prognosis factors for breast cancer. An OS-related nomogram was developed based on these independent prognosis factors and displayed good predicting ability for OS of breast cancer patients. Finally, our results revealed that KIF2C was significantly related to immune cell infiltration, tumor mutational burden, and immunotherapy in patients with breast cancer.

**Conclusion:**

KIF2C was overexpressed in breast cancer and was positively correlated with immune cell infiltration and immunotherapy response. Therefore, KIF2C can serve as a potential biomarker for prognosis and immunotherapy in breast cancer.

**Supplementary Information:**

The online version contains supplementary material available at 10.1186/s12885-023-10788-4.

## Introduction

Breast cancer is the most common cancer and the leading cause of cancer death in women all over the world. Globally, there are about 1.7 million breast cancer cases reported every year. The incidence of breast cancer is influenced by many factors, such as aging, breast characteristics, reproductive patterns, and genetic mutations [1, 2]. According to the molecular and histological features, breast cancer can be classified into several subtypes, including luminal A, luminal B, human epidermal receptor 2 overexpressing (HER2+), and triple negative (ER-, PR-, HER2-) breast cancer (TNBC). Luminal A and B subtypes are enriched with estrogen receptor positive (ER+) and/or progesterone receptor positive (PR+)[3, 4].

The therapeutic strategy should be made according to the molecular characteristics of breast cancer. For example, endocrine therapy is widely used for treating ER + breast cancer patients, while the combination of chemotherapy and anti-HER2 agents is the standard therapy for patients with HER2 + breast cancer. Chemotherapy is the first treatment option for patients with TNBC, due to lacking of therapeutic targets. Immunotherapy is emerging as a major therapeutic approach for breast cancer. It is generally believed that our immune system can recognize and kill cancer cells, but the functions and mechanisms of immune cells in cancer pathogenesis have not been fully understood. Different immune cells may exert distinct effects on cancer cells [5]. Natural killer (NK) cells, lymphocytes, and CD8 + T cells are capable of eradicating cancer cells [6–9]. The high tumor infiltrating lymphocytes (TILs) ratio determines the outcome of immunotherapy in breast cancer [10, 11]. HER2 + and TNBC breast cancer patients with high TILs usually have a better prognosis [12, 13]. On the contrary, some immune cells like T regulatory (Treg) cells, CD4 + T cells, and myeloid-derived suppressor cells (MDSCs) can promote tumor growth, and are associated with invasiveness and metastasis of breast cancer[14–16]. Accumulating evidence demonstrated that the development, progression, and relapse of many cancers are relevant to T cell dysfunction and immune checkpoint molecules-mediated immunosuppression. The immune checkpoint molecules include programmed death receptor 1 (PD-1), programmed cell death 1 ligand 1 (PD-L1), and cytotoxic T-lymphocyte-associated antigen 4 (CTLA-4). Recent cancer immunotherapy studies focus on immune checkpoint inhibitors (ICIs), such as anti-PD-1, anti-PD-L1, and anti-CTLA-4 antibodies. These checkpoint inhibitors can block immune inhibitory pathways that lead to tumor escape and enhance T cell immunity against tumors. The application of ICIs in TNBC has demonstrated improving outcomes in patients. Recently, PD-1-blockade has been approved for the treatment of stage II/III TNBC and PD-L1-positive TNBC patients with metastatic disease [17]. However, the currently known biomarkers, such as PD-1, PD-L1, and CTLA-4, are not effective for predicting the clinical efficacy of immunotherapy. Thus, there was great interest in the search for biomarkers that make accurate predictions on immunotherapy efficacy.

The kinesin-13 family member 2C (KIF2C), also known as mitotic centromere-associated kinesin (MCAK), belongs to the kinesin-13 family. In mammals, apart from the best characterized KIF2C, this family contains two other members, KIF2A and KIF2B. They share a very conversed kinesin motor domain in the middle of the polypeptide. Unlike other kinesins which move along microtubules (MTs) to transport cargos using energy from ATP hydrolysis, kinesin-13 proteins depolymerize MTs by disassembling tubulin subunits at the polymer ends [18]. Therefore, kinesin-13 proteins are involved in many MT-dependent activities, including mitosis and ciliogenesis. As KIF2C is the only kinesin protein that localizes to the centromere, it is proposed that KIF2C plays more important roles in maintaining genomic stability than other kinesin proteins. During mitosis, KIF2C is associated with centromere to ensure the proper kinetochore-microtubule attachments and thus correct chromosome segregation [18]. A recent study revealed that KIF2C could govern double-strand DNA break dynamics to enhance DNA damage repair and maintain genomic stability [19]. In addition, KIF2C can promote primary cilia disassembly through its microtubule depolymerizing activity, although this effect is less obvious compared to KIF2A [20].

Aberrant expression of KIF2C has been frequently associated with tumorigenesis. Up-regulation of KIF2C has been observed in various cancers, including breast cancer, hepatocellular carcinoma, colorectal cancer, gastric cancer, gliomas, endometrial cancer, and non-small cell lung cancer [21–28]. In hepatocellular carcinoma, KIF2C expression is up-regulated by Wnt/β-catenin signaling. Overexpression of KIF2C could promote cancer cell proliferation, migration, invasion, and metastasis by increasing the mTORC1 signaling transduction [21]. Moon et al. reported that KIF2C was able to control cancer cell migration and invasion through modulating MT dynamics and focal adhesion turnover [27]. Several previous studies have shown that KIF2C and other kinesins were overexpressed in breast cancer and associated with worse prognosis [29–33]. Furthermore, KIF2C has been reported to be correlated with immune cell infiltration in glioma, hepatocellular carcinoma and endometrial cancer [28, 34–36]. However, whether KIF2C has any effect on immunity response in breast cancer remains unknown.

Bioinformatics analysis is a powerful tool to analyze the roles and functional pathways of specific genes involved in cancer developments by using public databases, to give hints and predictions for cellular and in vivo research and clinical applications. This tool has been widely used in cancer research [37–40]. In this study, we conducted a serial of bioinformatics analysis to investigate the role of KIF2C in the occurrence and progression of breast cancer. We found that KIF2C is positively correlated with immune cell infiltration and immunotherapy response, suggesting that KIF2C might serve as a potential biomarker for prognosis and immunotherapy in breast cancer.

## Materials and Methods

### Data source

Transcriptome and clinical data of 899 breast cancer patients, including 881 patients with survival information and 90 normal tissues were downloaded from The Cancer Genome Atlas (TCGA) database (https://www.cancer.gov/tcga/). The GSE36295 dataset, including 5 normal and 45 breast cancer patients, the GSE42568 dataset, including 17 normal and 104 breast cancer patients with survival information, and the GSE20685 dataset, including 327 breast cancer patients with survival information, were downloaded from the Gene Expression Omnibus (GEO) database (http://www.ncbi.nlm.nih.gov/geo/).

### KIF2C expression analysis

Firstly, the Tumor Immune Estimation Resource (TIMER) database (http://timer.cistrome.org/) was used to investigate the expression of KIF2C in tumors and matched normal samples based on TCGA transcriptome data. Next, KIF2C mRNA expressions in 899 breast cancer patients and 90 normal tissues were analyzed. Additionally, the relationship between the expression level of KIF2C and clinical parameters, including age, TMN stage, N status, T status, and molecular subtypes, was investigated using the TCGA database.

### Immunohistochemistry

The formalin-fixed, paraffin-embedded human breast cancer tissue microarray slide (Cat# HBre-Duc060CS-04) was purchased from Shanghai Outdo Biotech Co., Ltd., Shanghai, China. The slide contained samples from 30 individual cases. Immunohistochemistry staining was performed as described previously [41]. Mouse anti-KIF2C (Invitrogen Cat# MA525647, 1:100) was used as the primary antibody. The stained slide was scanned using the Nikon automated whole slide scanning system (DS-U3). The expression level of KIF2C was assessed by the histochemistry score (H-score). Statistical significance was determined by paired t-test.

### Survival analysis

Following the different analysis of KIF2C expression between the case and control groups, the correlation between KIF2C expression and prognosis of breast cancer patients was analyzed according to previous studies [37, 39]. Breast cancer patients with survival information in the TCGA and the GSE42568 dataset were divided into the high and low expression groups based on the median expression of KIF2C, separately. Overall survival (OS) was explored by Kaplan-Meier survival curves through the “survival” R package. Furthermore, stratified survival analysis was performed to observe the OS among different clinical parameters using the “Survminer 0.4.6” R package.

### Independently prognostic analysis

Firstly, univariate Cox was used to screen clinical parameters and KIF2C expression related to the prognosis of breast cancer patients. Next, multivariate Cox analysis was used to identify independent prognostic factors. Moreover, the “rms 5.1-4” R package was used to create a nomogram to guide clinical practice of the breast cancer patients. C-index, calibration curve, the area under the curve (AUC) as well as decision curve analysis (DCA) curves were used to assess the predictive efficiency and clinical utilize of the nomogram, in which ROC and DCA curves were drawn by the “pROC 1.16.2” package and the “magick 2.7.2” package, respectively.

### Gene set enrichment analysis (GSEA)

Single gene GSEA was performed to investigate the Gene Ontology (GO) functions and Kyoto Encyclopedia of Genes and Genomes (KEGG) pathways [42–44] related to KIF2C through the clusterProfiler 3.16.0 R package. Firstly, we calculated the expression correlation co-efficiency between KIF2C and other genes. Next, the gene expression matrix ranked by correlation co-efficiency was used to perform GSEA. Moreover, single-sample GSEA (ssGSEA) was used to calculate the enrichment scores of the top 10 GO and KEGG terms by GSVA 1.36.2 R package.

### Immune infiltration and immunotherapy efficiency analysis

The immune score, stromal score, and estimate score were calculated by Estimation of Stromal and Immune cells in Malignant Tumor tissues using the Expression (ESTIMATE) algorithm via the ESTIMATE 1.0.13 R package. These scores were used to assess the difference in tumor microenvironment between the high and low KIF2C expression groups. In addition, the microenvironment cell population-counter (MCP-counter) was used to assess the composition of immune cells, epithelial cells, and fibroblasts in the high and low KIF2C expression groups by MCP-counter 1.2.0 R package. Besides, ssGSEA was used to calculate the enrichment scores of 28 immune-related gene sets in the high and low KIF2C expression groups. Cytolytic activity is a biomarker of antitumor immunity and prognosis in cancer [45]. Therefore, we further compared the difference in cytolytic activity scores between the high and low KIF2C expression groups based on gene expression profiles of effector proteins granzyme and perforin. Furthermore, we investigated the tumor mutational burden in the high and low KIF2C expression groups. The expression difference of immune checkpoint molecules between the high and low KIF2C expression groups was compared. Finally, the Tumor Immune Dysfunction and Exclusion (TIDE) algorithm was selected to assess the clinical response to immunotherapy in the high and low KIF2C expression groups.

### Construction of a competing endogenous RNA (ceRNA) network

In order to investigate the potential regulatory mechanism in breast cancer, the KIF2C-related ceRNA network was predicted through Miranda 3.3a software [46]. The potential miRNAs targeting KIF2C were firstly predicted with the cut-off of binding score > 500 and free energy score < -500. Similarly, the predictive miRNAs-related lncRNAs were further exhibited with the cut-off of binding score > 500 as well. Finally, a ceRNA network was illustrated by Cytoscape 3.7.2.

### Statistical analysis

R programming language (version 4.0.2) and corresponding packages were utilized to perform statistical analyses and visualization. Wilcoxon rank-sum test and paired t-test were used to estimate the differences between two groups. *P* < 0.05 was considered significant. The *P* values were indicated as: *P < 0 0.05, **P < 0 0.01, ***P < 0.001, ****P < 0.0001, ns, not significant.

## Results

### KIF2C is upregulated in breast cancer

**Fig. 1:**
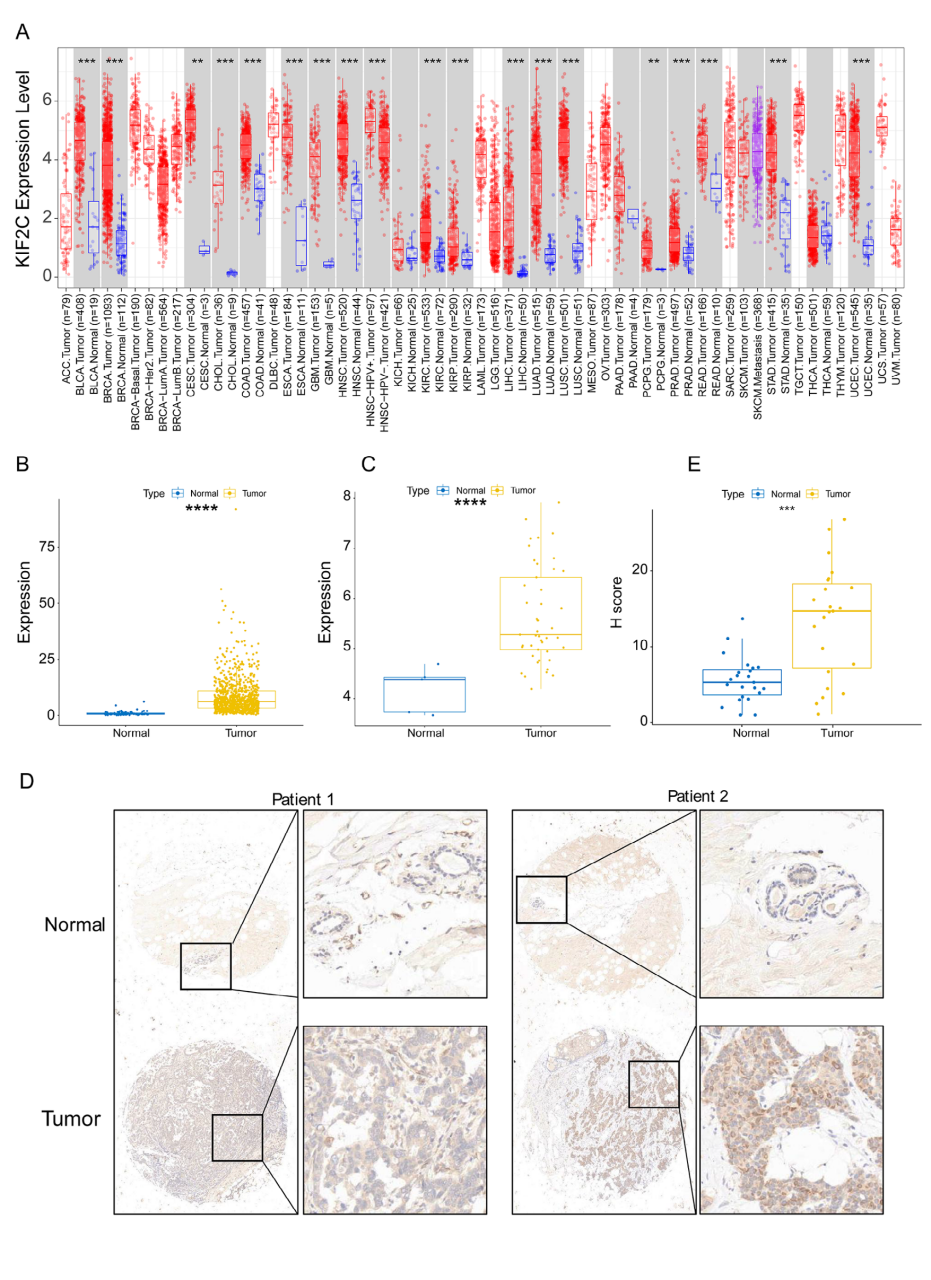
Upregulation of KIF2C expression in breast cancer. (A) The expression of KIF2C in paired tumor and normal tissue samples in TIMER database. (B) The expression of KIF2C between breast cancer and adjacent normal tissue samples in the TCGA database. (C) The expression of KIF2C between breast cancer and adjacent normal tissue samples on the GSE36295 dataset. (D) Representative images of immunohistochemical staining of KIF2C in breast cancer and paired adjacent normal tissues. The expression of KIF2C is upregulated in breast cancer tissues, as compared to their paired non-tumor tissues. (E) Box plot showing H-scores on tissue array for both tumor tissues and paired non-tumor tissues. *P < 0 0.05, **P < 0 0.01, ***P < 0.001, ****P < 0.0001

The mRNA expression levels of KIF2C in tumor samples and matched normal samples were investigated using the TIMER database. The results showed that the mRNA expression of KIF2C was remarkably upregulated in 18 human cancer tissues compared with matched normal tissues, including breast cancer, bladder urothelial carcinoma (BLCA), cervical squamous cell carcinoma and endocervical adenocarcinoma (CESC), cholangiocarcinoma (CHOL), colon adenocarcinoma (COAD), esophageal carcinoma (ESCA), glioblastoma multiforme (GBM), head and neck squamous cell carcinoma (HNSC), kidney chromophobe (KIRC), kidney renal papillary cell carcinoma (KIRP), liver hepatocellular carcinoma (LIHC), lung adenocarcinoma (LUAD), lung squamous cell carcinoma (LUSC), pheochromocytoma and paraganglioma (PCPG), prostate adenocarcinoma (PRAD), rectum adenocarcinoma (READ), stomach adenocarcinoma (STAD), and uterine corpus endometrial carcinoma (UCEC) **(**Fig. 1A**)**. We further examined KIF2C expression in breast cancer and matched normal tissues using downloaded data from the TCGA database and the GSE36295 dataset. Consistent with the results from the TIMER database, a significantly higher level of KIF2C expression was observed in breast cancer tissues than in normal tissues in both the TCGA database and the GSE36295 dataset **(**Fig. 1B, C**)**. These results demonstrated that KIF2C is upregulated in breast cancer.

To verify the expression pattern of KIF2C protein in breast cancer, an immunohistochemistry analysis was performed on paraffin-embedded breast cancer tissue microarray. The results showed that, in agreement with our bioinformatic analysis, KIF2C was upregulated in breast cancer tissues, as compared to the adjacent normal tissues **(**Fig. 1D, E**)**.

### KIF2C expression is associated with the progression of breast cancer

**Fig. 2:**
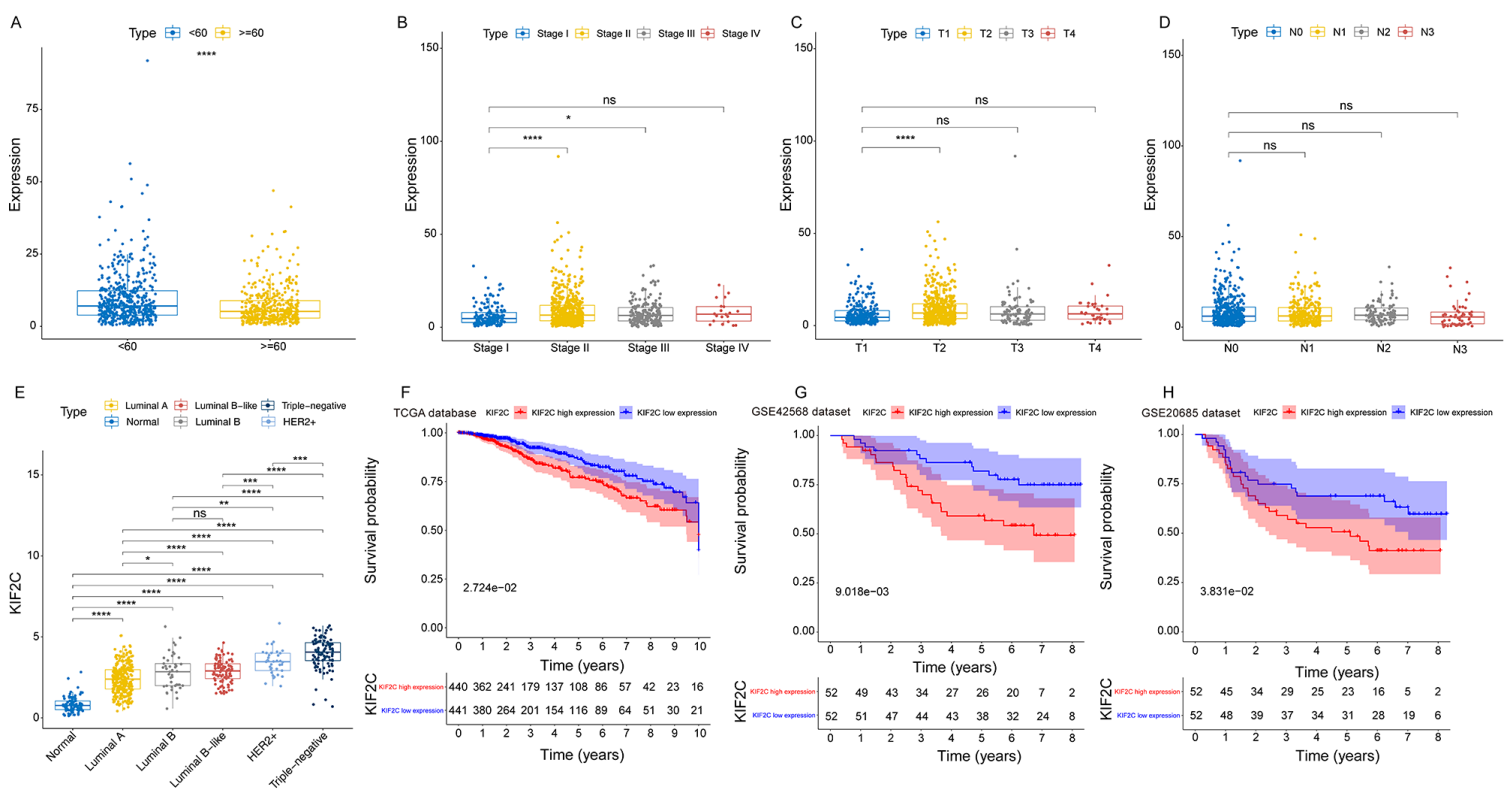
Correlation between the expression of KIF2C and clinical pathological characteristics of patients with breast cancer: Age (A), TMN stage (B), T status (C), N status (D), molecular subtypes (E). (F-H) The effects of different expression levels of KIF2C on the OS of patients with breast cancer using TCGA database (F), GSE42568 datasets (G) and GSE20685 datasets (H). *P < 0 0.05, **P < 0 0.01, ***P < 0.001, ****P < 0.0001, ns, not significant

To evaluate the role of KIF2C in breast cancer, the relationship between clinical parameters (including age, tumor stage, and tumor TNM stage) and KIF2C expression was analyzed using data from the TCGA database. We found that the expression of KIF2C was significantly elevated in breast cancer patients under 60 years of age compared with those over 60 years of age **(**Fig. 2A**).** Furthermore, KIF2C expression in breast cancer patients with stage II and III was higher than that in breast cancer patients with stage I **(**Fig. 2B**).** In addition, KIF2C was upregulated in breast cancer patients with stage T2 compared with breast cancer patients with stage T1 **(**Fig. 2C**).** However, there was no significant difference in KIF2C expression between breast cancer patients with different stages N **(**Fig. 2D**)**. KIF2C expression in breast cancer patients with different molecular subtypes was also examined. The results showed that KIF2C was upregulated in all subtypes of breast cancer. Among different subtypes, triple-negative breast cancer showed highest KIF2C expression while luminal A subtype had lowest expression **(**Fig. 2E**)**. Collectively, these data suggested that KIF2C expression may be influenced by age and involved in the progression of breast cancer.

To investigate whether KIF2C expression is associated with the prognosis of breast cancer, Kaplan-Meier survival analysis was performed using downloaded data from the TCGA database, GSE20658, and GSE42568 datasets. We found that higher KIF2C expression was associated with poor OS in breast cancer (Fig. 2F H). We further performed a univariate Cox analysis to determine whether KIF2C was an independent OS factor in breast cancer. As shown in Tables 1and Supplementary Fig. 1A, KIF2C expression was significantly correlated to OS in breast cancer patients (HR = 1.56607088, *P* = 0.01784196). We noted that the OS of breast cancer patients was also affected by age (1.03485369, *P* = 7.23E-06), tumor stage (HR = 2.28016073, *P* = 2.95E-11), stage T (HR = 1.58856729, *P* = 4.54E-05), stage N (HR = 1.69043662, *P* = 5.01E-08), stage M (HR = 6.86369543, *P* = 8.21E-11), and estrogen receptor (ER, HR = 0.62096529, *P* = 0.01822903). Furthermore, risk parameters for OS identified by univariate Cox analysis were subjected to multivariate Cox regression analysis. Our results showed that age (HR = 1.04341252, *P* = 3.17E-08), TNM stage (HR = 2.39546349, *P* = 8.93E-13), ER (HR = 0.58848779, *P* = 0.01866529), and high KIF2C expression (HR = 1.60169321, *P* = 0.02724886) were independent prognostic factors for OS in breast cancer patients (Tables 2 and Supplementary Fig. 1B).

**Table 1:** Univariate Cox regression analysis for OS of patients with breast cancer

Variation	Coeff	HR	HR.95 L	HR.95 H	P value
Age	0.03426005	1.03485369	1.01948169	1.05045746	7.23E-06
Stage	0.82424593	2.28016073	1.78831559	2.90727931	2.95E-11
M	1.92624599	6.86369543	3.83864517	12.2726412	8.21E-11
N	0.52498685	1.69043662	1.39964552	2.04164264	5.01E-08
T	0.46283254	1.58856729	1.27176281	1.98428987	4.54E-05
KIF2C	0.44856986	1.56607088	1.08050916	2.26983546	0.01784196
ER	-0.47648008	0.62096529	0.41809894	0.92226472	0.01822902
Gender	-0.11483671	0.89151171	0.12414484	6.40214373	0.90910579

**Table 2:** Multivariate Cox regression analysis for OS on patients with breast cancer

ID	Coeff	HR	HR.95 L	HR.95 H	P value
Age	0.04249661	1.04341252	1.0278198	1.05924179	3.17E-08
Stage	0.87357674	2.39546349	1.88509624	3.04400657	8.93E-13
ER	-0.53019911	0.58848779	0.3783273	0.91539223	0.01866529
KIF2C	0.471061323	1.60169321	1.05432874	2.43322697	0.02724886

**Fig. 3:**
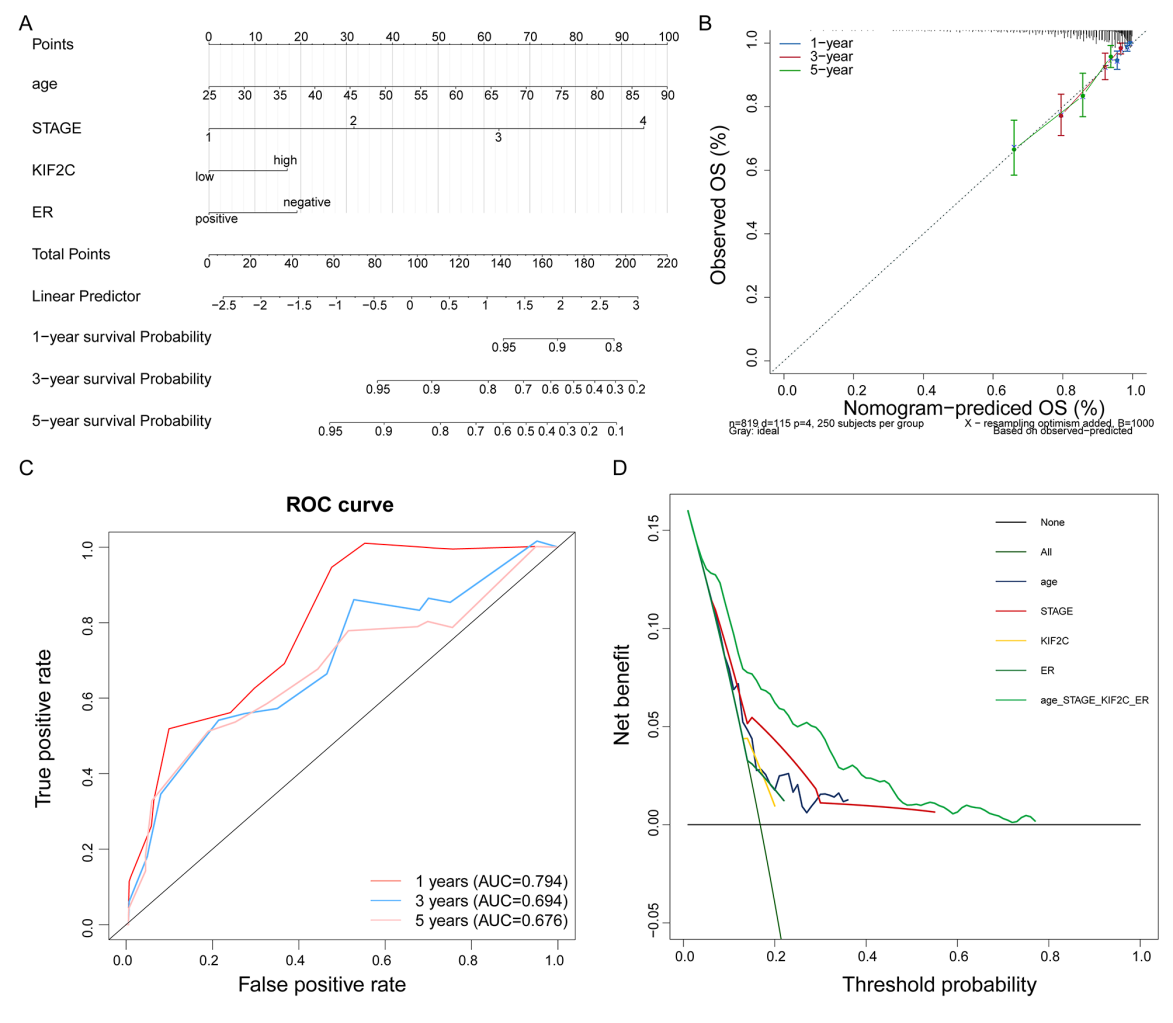
Construction and evaluation of nomogram for OS prediction of patients with breast cancer. (A) Nomograms predicting 1-year, 3-year and 5-year survival of breast cancer patients based on age, TMN stage, ER and KIF2C expression. (B) Calibration curve of nomogram. (C) ROC curve of nomogram. (D) Decision curve analysis (DCA) curve for the clinical utilize of nomogram

Therefore, a nomogram predicting 1-year, 3-year, and 5-year survival of breast cancer patients was constructed based on independent prognostic factors selected by multivariate Cox regression analysis (Fig. 3A). The C-index of the nomogram for predicting the 1-year, 3-year, and 5-year OS was 0.9465, 0.7839, and 0.6845, respectively. The calibration plots presented excellent consistency between the probabilities predicted by the nomograms and the actual observations in terms of 1-year, 3-year, and 5-year OS of the TCGA cohort **(**Fig. 3B). The results of the ROC analysis demonstrated that the areas under the ROC curves for predictive 1-year, 3-year, and 5-year OS of the nomogram were 0.794, 0.694, and 0.676, respectively **(**Fig. 3C). The results of the decision curve analysis showed that the nomogram was able to better predict breast cancer prognosis than a single factor **(**Fig. 3D). Overall, these results illustrated that the nomogram could be used as an effective method to predict the prognosis of patients with breast cancer.

### Identification of KIF2C-related biological functions

**Fig. 4:**
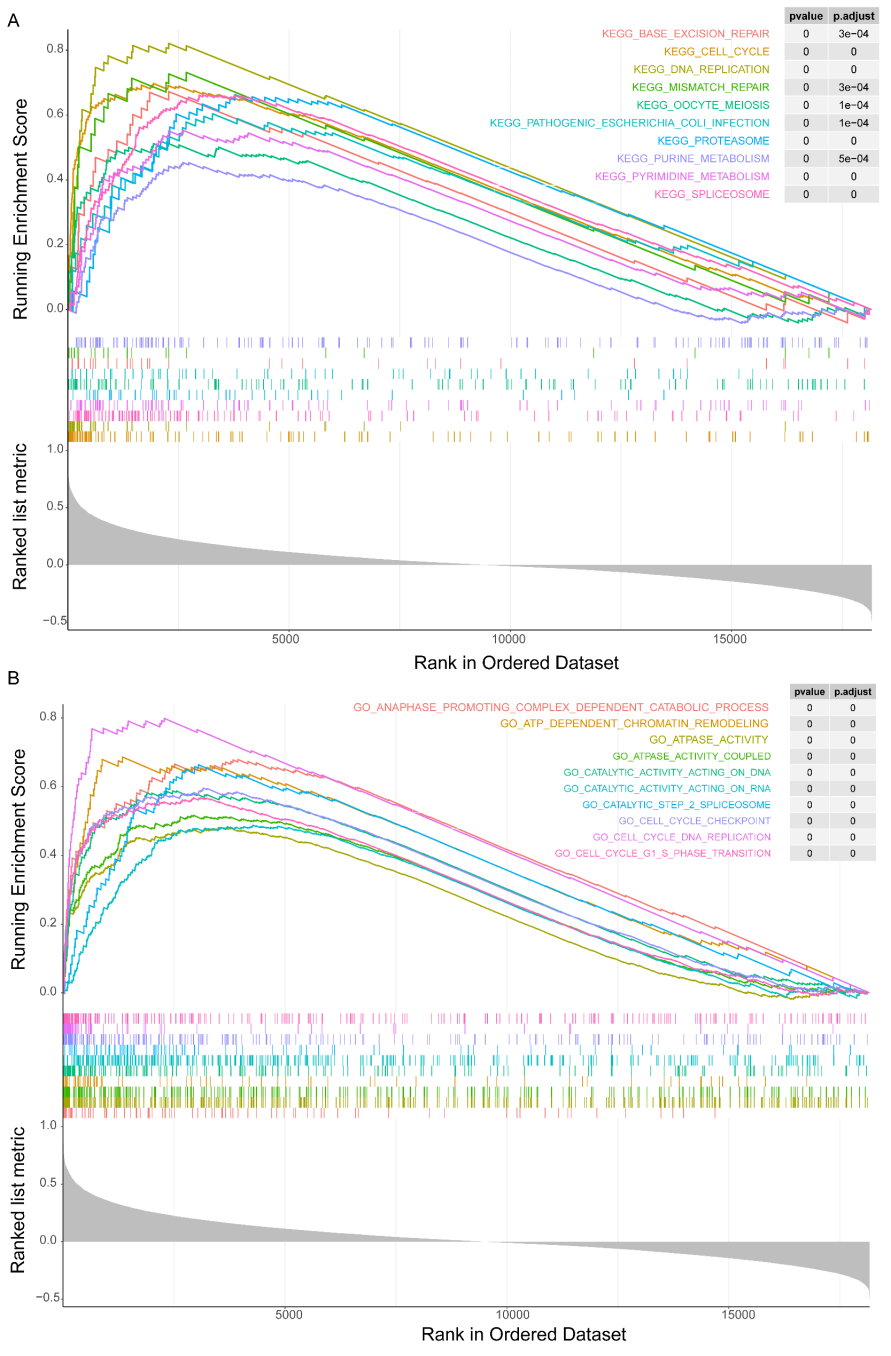
Biological processes involved in KIF2C interactive genes. The Gene Ontology (GO) (A) and Kyoto Encyclopedia of Genes and Genomes (KEGG) (B) enriched pathways related to KIF2C was investigated using Single Gene GSEA analysis. *P < 0.05

To explore the potential biological functions of KIF2C, GSEA was performed based on the expression matrix of genes related to KIF2C. The KEGG pathway showed that the KIF2C-related genes were involved in the cell cycle, base excision repair, DNA replication, and purine and pyrimidine metabolism **(**Fig. 4A**)**. The functions of these genes were mainly associated with anaphase promoting complex-dependent catabolic process, ATP-dependent chromatin remodeling, catalytic activity acting on DNA, ATPase activity, and the cell cycle-related functions (Fig. 4B). Furthermore, we found that the enrichment scores of the top 10 GO functions and KEGG pathways involved in KIF2C-related genes were different between the high and low KIF2C expression groups, and the enrichment scores of the top 10 GO functions and KEGG pathways were higher in high-KIF2C expression group (Supplementary Fig. 2A and 2B). Thus, we speculated that the expression and function of KIF2C-related genes might be associated with poor prognosis in breast cancer patients with high expression of KIF2C.

### KIF2C is correlated with immune cell infiltration and immunotherapy in breast cancer patients

**Fig. 5:**
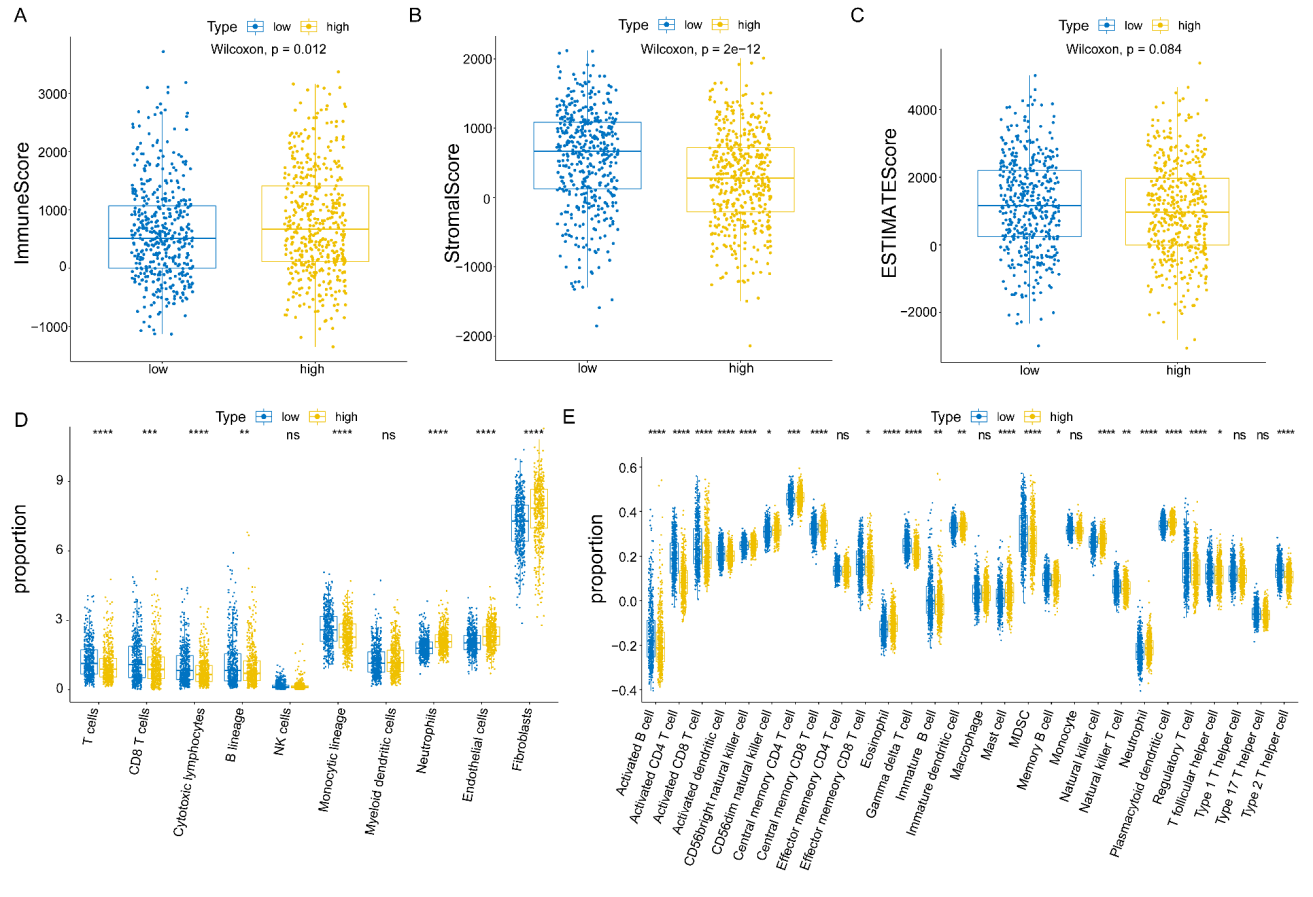
Correlation between KIF2C expression and immune cell infiltration in breast cancer. (A-C) Immune scores, stromal scores and ESTIMATE scores (P = 0.084) between high KIF2C group and low KIF2C group, respectively. The abundance of various cells in the tumor microenvironment analyzed by MCP-counter algorithm (D) and ssGSEA (E).*P < 0 0.05, **P < 0 0.01, ***P < 0.001, ****P < 0.0001, ns, not significant

To determine the correlation between KIF2C and tumor microenvironment, we firstly compared the differences in immune scores, stromal scores, and ESTIMATE scores between the high and low KIF2C expression groups. We found significantly increased immune scores in the high KIF2C expression group compared with the low expression group **(**Fig. 5A**)**, whereas stromal scores dramatically decreased in the high KIF2C expression group compared with the low expression group **(**Fig. 5B**)**. However, the ESTIMATE scores between the high and low KIF2C expression groups were not significantly different **(**Fig. 5C**)**. Moreover, the MCP-counter algorithm and ssGSEA analyses were performed to compare the difference in infiltrating immune cells in the tumor microenvironment of breast cancer patients. The results of MCP-counter showed an increased number of tumor infiltrating immune cells, including T cells, CD8 T cells, cytotoxic lymphocytes, monocytic lineage, and neutrophils in the high KIF2C expression group compared with the low expression group, while the number of endothelial cells and fibroblasts was significantly higher in the low KIF2C group **(**Fig. 5D, Supplementary Fig. 3A). Consistently, the results of ssGSEA also revealed that more infiltrating immune cells were detected in the high KIF2C expression group compared with the low expression group **(**Fig. 5E, Supplementary Fig. 3B).

**Fig. 6:**
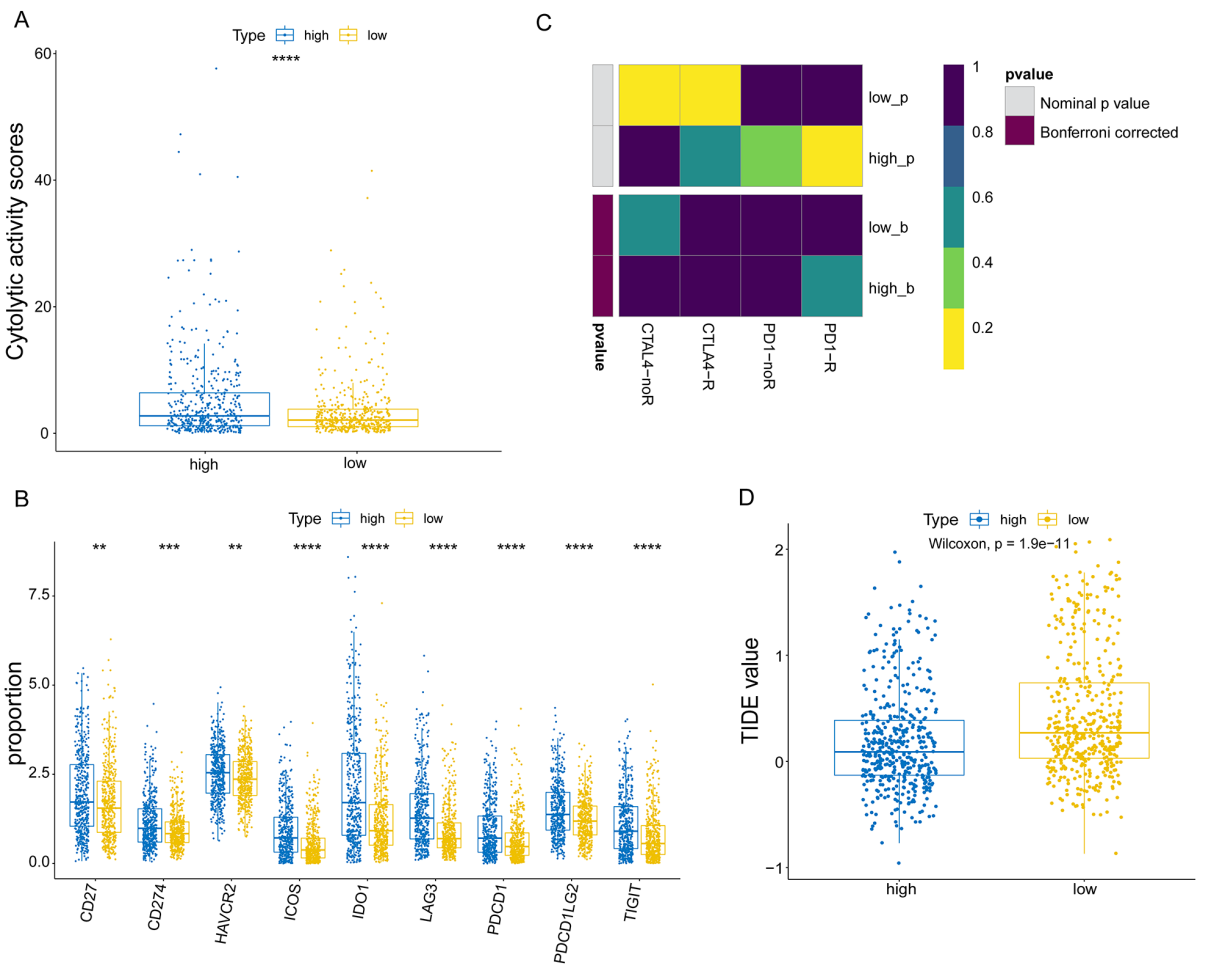
Correlation between KIF2C expression and immunotherapy in breast cancer. (A) Cytolytic activity scores between high and low KIF2C expression group. (B) The expression of the immune checkpoint molecules between high and low KIF2C group. (C) Heatmap for predicting effect on anti-PD-1 and anti-CTLA4-R therapy. (D) TIDE immunotherapy prediction analyses between high and low KIF2C group. *P < 0 0.05, **P < 0 0.01, ***P < 0.001, ****P < 0.0001

In addition, we found that patients in the high KIF2C expression group presented higher cytolytic activity scores compared with those in the low expression group **(**Fig. 6A**)**, suggesting increased cytotoxic T cell infiltration in the high KIF2C expression group.

We further analyzed the expression of immune checkpoint molecules in the low and high KIF2C expression groups. We found that only PDCD1 and PDCD1LG2 showed higher expressions in the low KIF2C expression group compared with the high expression group, whereas CD27, CD274, HAVCR2, ICOS, IDO1, LAG3, and TIGIT exhibited higher expressions in the high KIF2C expression group **(**Fig. 6B**)**. The results of tumor mutational burden analysis also showed that breast cancer patients in the high KIF2C expression group had a higher tumor mutational burden compared with the low expression group **(**Fig. 6C**)**. Additionally, the TIDE algorithm was used to predict the efficacy of immunotherapy for breast cancer patients in the high and low KIF2C expression groups. As shown in Fig. 6D, the TIDE score was significantly lower in the high KIF2C expression group compared with the low expression group, suggesting lower immune escape potential in the high expression group. Together, these discoveries revealed that high expression of KIF2C may be associated with the higher immune cell infiltration, higher tumor mutational burden, and better response to immunotherapy in breast cancer.

### Construction of the KIF2C-related ceRNA network

**Fig. 7:**
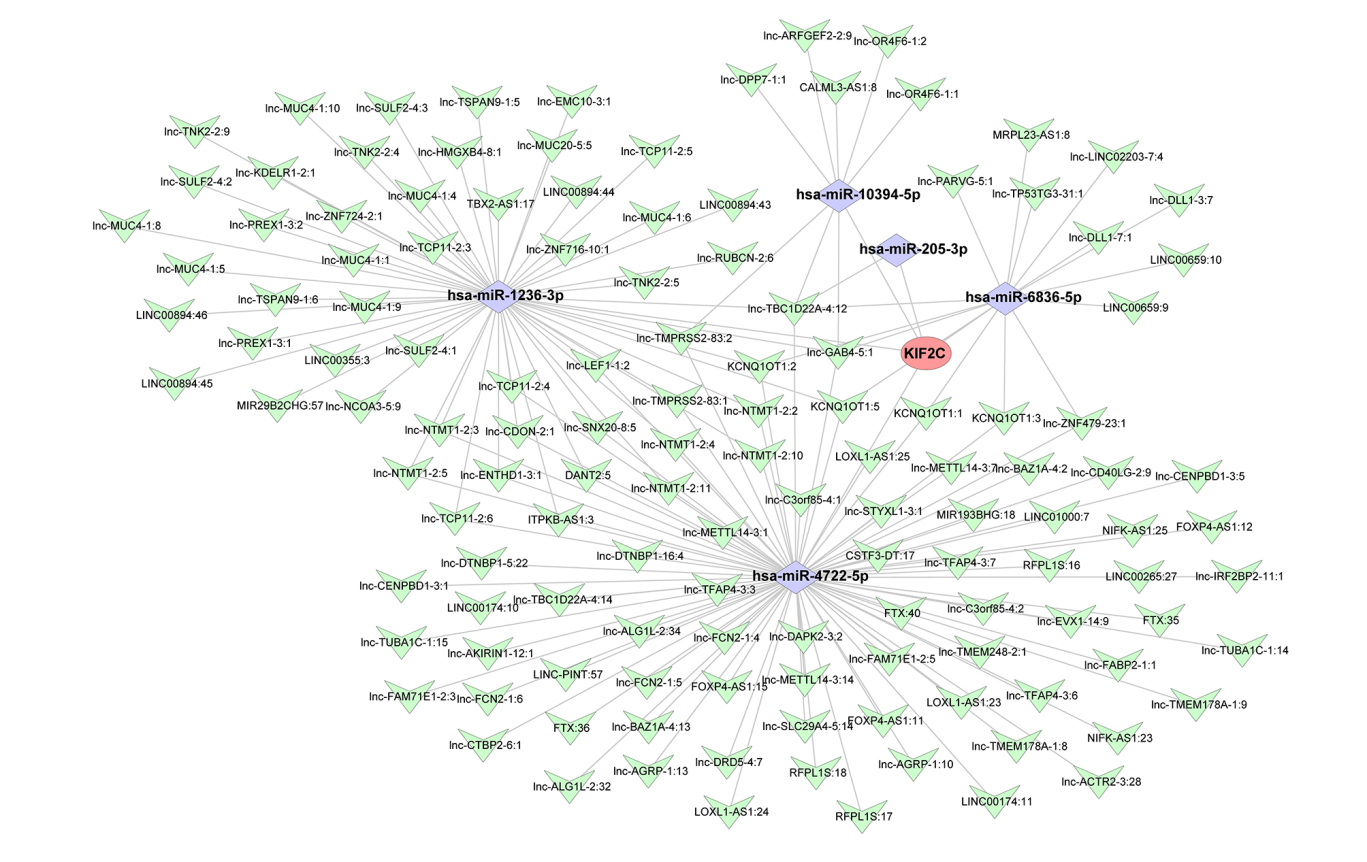
The competing endogenous RNA (ceRNA) network targeting the KIF2C gene through Miranda 3.3a software. Red oval represents KIF2C gene, purple diamond represents miRNA, and green V-shape represents lncRNA.

To explore the potential regulatory mechanisms of KIF2C expression in breast cancer, we constructed a KIF2C-related ceRNA network **(**Fig. 7**)**. As shown in Fig. 7, the expression of KIF2C might be modulated by hsa-miR-10394-5P, hsa-miR-1236-3P, hsa-miR-205-3P, hsa-miR-4722-5P, and hsa-miR-6836-5P. Moreover, 151 lncRNAs might be involved in the regulation of these miRNAs.

## Discussion

In the present study, KIF2C was found to be up-regulated in breast cancer in all subtypes of breast cancer compared to normal tissues. Among different subtypes, triple-negative subtype expressed the highest KIF2C, while luminal A subtype had the lowest expression. Compared to luminal A subtype, the other four subtypes of breast cancer, luminal B, luminal B-like, HER2 + and triple-negative breast cancer are more malignant and associated with poor prognosis [47]. Our data suggest that KIF2C may be involved in the development of higher grade of breast cancer, and a high level of KIF2C is significantly associated with poor OS in breast cancer patients. Importantly, our results demonstrated that KIF2C may serve as an independent prognostic factor for breast cancer, which is different from age, tumor size, tumor stage, and other traditional biomarkers.

The infiltrating immune cells are vital components of the tumor microenvironment and greatly influence patient prognoses and immunotherapy outcomes. Tumor-infiltrating lymphocytes (TILs) have been shown to be associated with the improved clinical outcomes of immunotherapy in melanoma, colorectal cancer, and ovarian cancer [11, 48]. In HER2 + and basal-like breast cancer patients, high levels of TILs are also correlated with a good prognosis [10, 14]. In this study, we provided several lines of evidence to demonstrate a significant positive correlation between KIF2C expression and the infiltrating immune cells in breast cancer. First, we found an increased number of tumor infiltrating immune cells, including T cells, CD8 + T cells, cytotoxic lymphocytes, monocytic lineage, and neutrophils in the high KIF2C expression group compared with the low expression group. These immune cells can enhance anti-tumor immune response and inhibit tumor growth [7, 8, 48–52]. Second, the ssGSEA analysis showed that more infiltrating immune cells were detected in the high KIF2C expression group compared with the low expression group. Third, our results demonstrated that patients in the high KIF2C expression group presented higher cytolytic activity scores, suggesting increased cytotoxic T cell infiltration. In addition, we found that high expression of the KIF2C gene was associated with a higher tumor mutational burden and a favorable response to immunotherapy in breast cancer. Increasing evidence demonstrated that HER2 + and TNBC breast cancer patients had increased TILs ratio, indicating a favorable prognosis [12, 13]. Our results revealed that KIF2C could act as a potential biomarker for prognosis and immunotherapy of breast cancer. Consistently, KIF2C has been shown to be positively associated with immune cell infiltration in glioma and hepatocellular carcinoma [34–36]. In contrast, the negative effect of KIF2C on immune cells infiltration has been observed in endometrial cancer [28]. Together with results from other studies, our data suggest that the function of KIF2C in immune cells infiltration may be cancer type-specific.

KIF2C is a microtubule-based motor protein and has multiple cellular regulatory functions, such as the regulation of mitosis, spindle assembly, microtubule depolymerization, chromosome congression and segregation, kinetochore-microtubule attachments, and genome stability [18]. As a mitotic centromere-associated kinesin, KIF2C has a more important role in genomic stability. KIF2C also play a role in primary cilia disassembly. Primary cilia are microtubule-based, antenna-like organelles that protrude from the plasma membranes [53]. The major function of primary cilia is to sense the extracellular signal and transduce it into cells [53]. Dysfunction of primary cilia leads to a wide range of diseases, including cancer [54]. Similar to other types of cancer, primary cilia are lost in breast cancer, which is an early event of tumorigenesis [55]. Up-regulation of KIF2C can promote the disassembly of primary cilia through its MT depolymerizing activity [20], which may contribute to the pathogenesis of breast cancer. Moreover, overexpression of KIF2C could promote cancer cell proliferation, migration, invasion, and metastasis by increasing the mTORC1 and MEK/ERK signaling transduction and modulating MT dynamics and focal adhesion turnover [21, 22, 27]. Consistent with previous studies [56, 57], our GSEA data also suggested that KIF2C is involved in the regulation of DNA replication and cell cycle control, which might be also responsible for its role in cancer development. Dysfunction of KIF2C may result in erroneous kinetochore-microtubule attachments, leading to the formation of lagging chromosomes and micronuclei. All of these, on one hand, would promote cancer progression. However, on the other hand, deregulation of KIF2C would also result in genomic instability, manifesting as the formation of micronuclei and cytosolic DNA breaks/fragments. Micronuclei and cytosolic DNA breaks/fragments can be detected by cGAS, a cytosolic DNA sensor [58]. After sensing cytosolic DNA, cGAS generates the second messenger 2’,3’-cGAMP, to phosphorylate STING. Activated STING further stimulates the expression of type I interferon genes and recruits T/NK cells to tumor microenvironment [59, 60].

## Conclusions

In summary, the bioinformatic analysis showed that KIF2C was upregulated in breast cancer and other types of cancer, and the high KIF2C expression was associated with poor prognosis in breast cancer. We further demonstrated that the expression of KIF2C was positively associated with immune cell infiltration of the tumor microenvironment, suggesting that KIF2C may act as a potential prognostic biomarker for breast cancer immunotherapy. Further in vitro and in vivo studies are needed to elucidate the role of KIF2C in breast cancer immune cell infiltration in the future.

## Electronic supplementary material

Below is the link to the electronic supplementary material.

Supplementary Material 1

## Data Availability

The datasets analyzed in the current study are available in The Cancer Genome Atlas (TCGA) database (https://www.cancer.gov/tcga/) and the Gene Expression Omnibus (GEO) database (http://www.ncbi.nlm.nih.gov/geo/).

## Abbreviations

ER+: Estrogen receptor positive
PR+: Progesterone receptor positive
HER2+: Human epidermal receptor 2 positive
TNBC: Triple negative breast cancer
NK cells: Natural killer cells
TILs: Tumor infiltrating lymphocytes
Treg cells: T regulatory cells
MDSCs: Myeloid-derived suppressor cells
PD-1: Programmed death receptor 1
PD-L1: programmed cell death 1 ligand 1
CTLA-4: Cytotoxic T-lymphocyte-associated antigen 4
ICIs: Immune checkpoint inhibitors
KIF2C: Kinesin-13 family member 2 C
MCAK: Mitotic centromere-associated kinesin
MTs: Microtubules
TCGA: The Cancer Genome Atlas
GEO: Gene Expression Omnibus
TIMER: Tumor Immune Estimation Resource
OS: Overall survival
GSEA: Gene set enrichment analysis
GO: Gene Ontology
KEGG: Kyoto Encyclopedia of Genes and Genomes
ssGSEA: single-sample GSEA
ESTIMATE: Estimation of Stromal and Immune cells in Malignant Tumor tissues using the Expression
MCPcounter: Microenvironment cell population-counter
TIDE: Tumor Immune Dysfunction and Exclusion
BLCA: Bladder urothelial carcinoma
CESC: Cervical squamous cell carcinoma and endocervical adenocarcinoma
CHOL: Cholangiocarcinoma
COAD: Colon adenocarcinoma
ESCA: Esophageal carcinoma
GBM: Glioblastoma multiforme
HNSC: Head and neck squamous cell carcinoma
KIRC: Kidney chromophobe
KIRP: Kidney renal papillary cell carcinoma
LIHC: Liver hepatocellular carcinoma
LUAD: Lung adenocarcinoma
LUSC: Lung squamous cell carcinoma
PCPG: Pheochromocytoma and paraganglioma
PRAD: Prostate adenocarcinoma
READ: Rectum adenocarcinoma
STAD: Stomach adenocarcinoma
UCEC: Uterine corpus endometrial carcinoma
