# Supplementary material for: KIF2C is a prognostic biomarker associated with immune cell infiltration in breast cancer
Source: BMC Cancer. 2023 Apr 4;23:307. doi: 10.1186/s12885-023-10788-4 (PMC10071625; doi:10.1186/s12885-023-10788-4)
Supplement: Supplementary file 1 — Supplementary Material 1 [file 12885_2023_10788_MOESM1_ESM.docx]

**Supplementary materials**

**
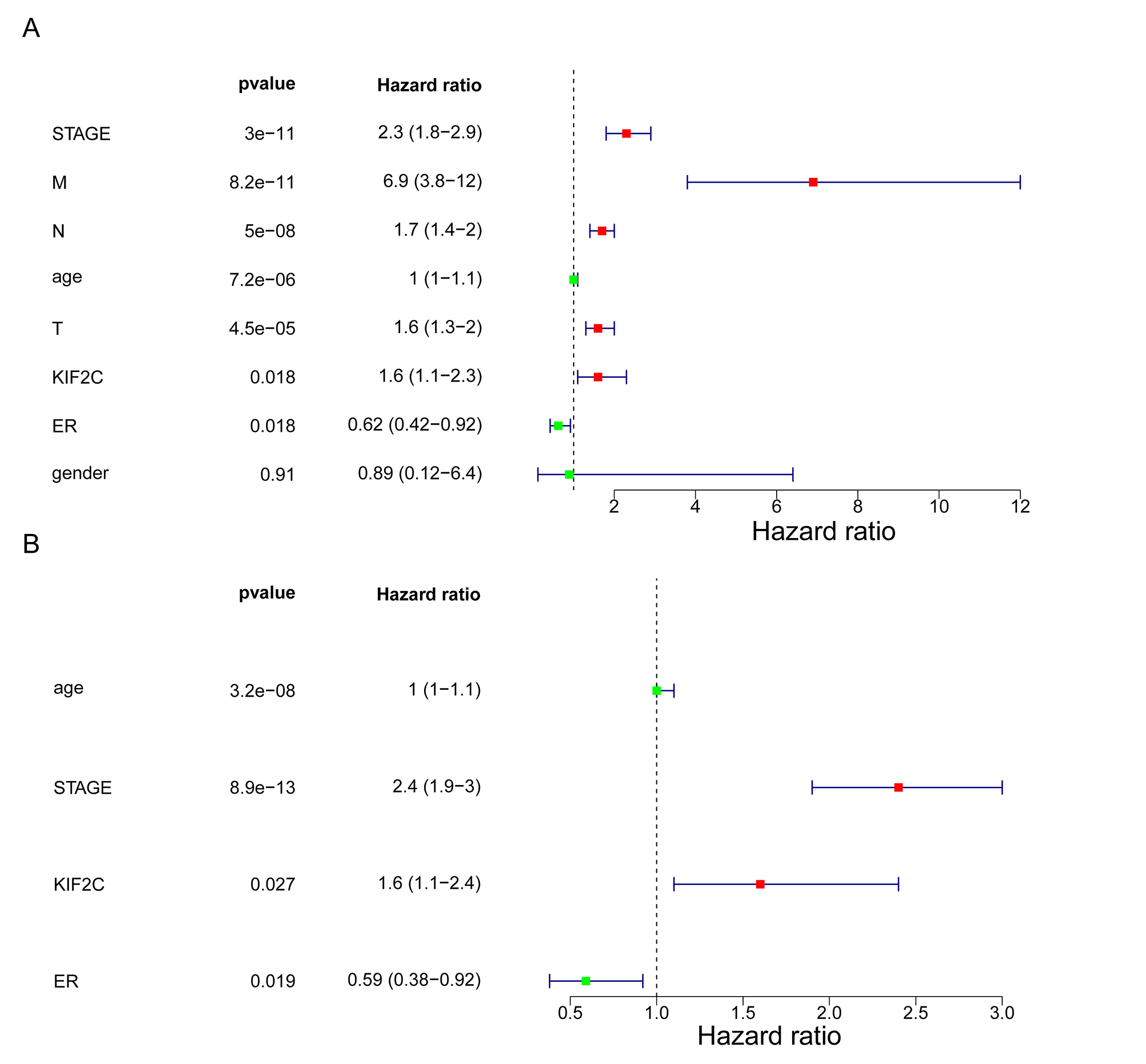
**

**Supplementary Figure 1.** Univariate (A) and multivariate (B) Cox regression analysis of OS-related factors in breast cancer.


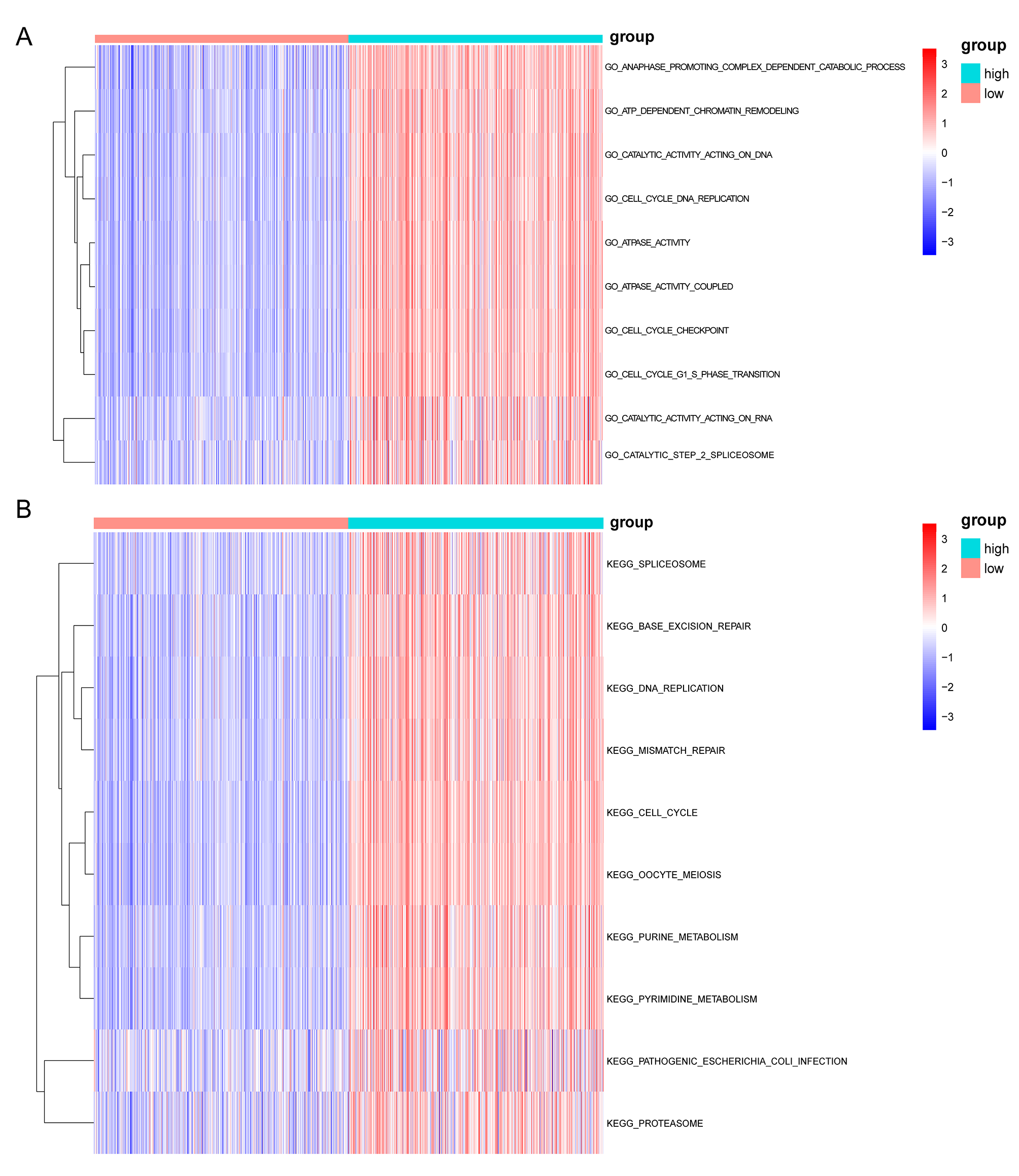


**Supplementary Figure 2.** Heatmaps for GO (A) and KEGG (B) enriched pathways using GSVA analysis between high and low KIF2C expression groups.


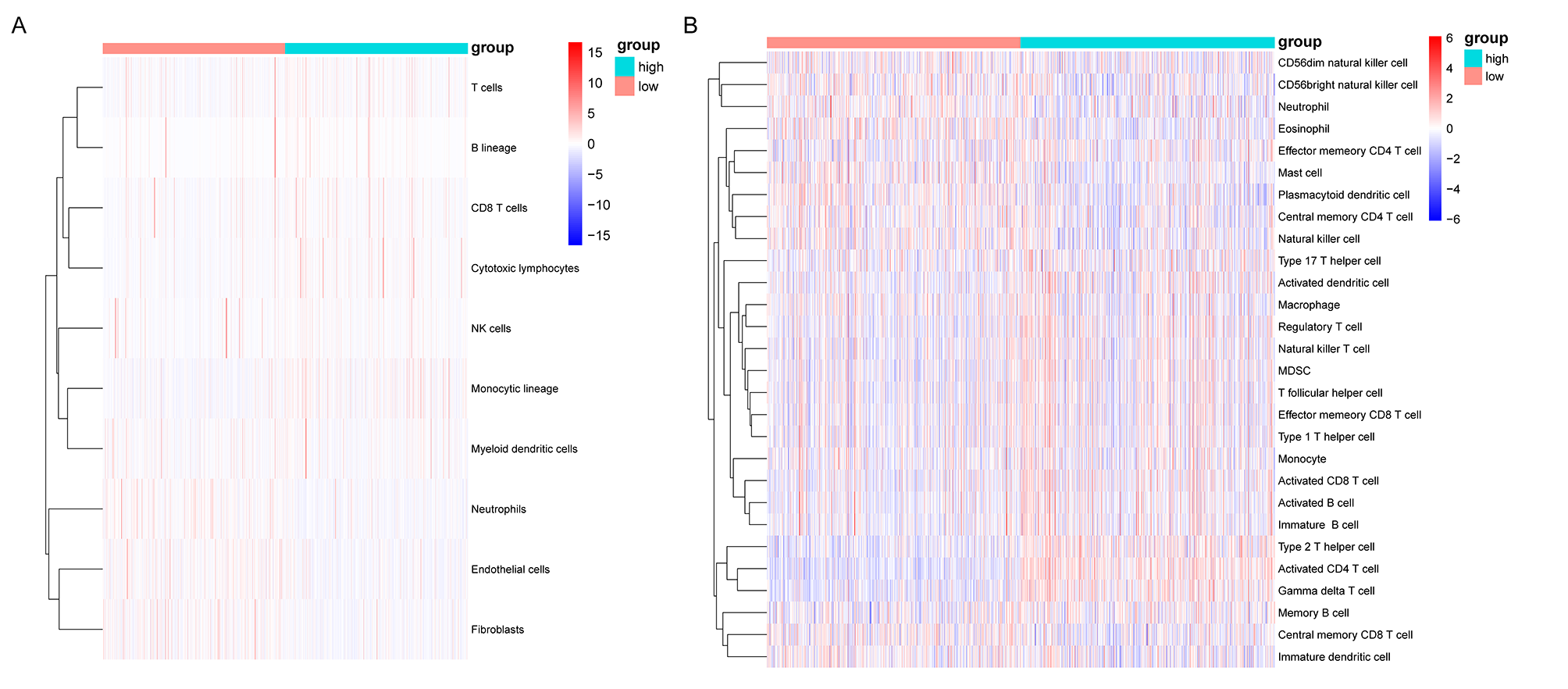


**Supplementary Figure 3.** (A) The abundance of different cell types in the high and low KIF2C expression groups calculated by MCP-counter. (B) The abundance of various cells in high and low KIF2C expression groups analyzed by ssGSEA.
